# Potentiating effect of imidacloprid on arsenic-induced testicular toxicity in Wistar rats

**DOI:** 10.1186/s40360-018-0239-9

**Published:** 2018-07-31

**Authors:** Lakshay Mahajan, Pawan Kumar Verma, Rajinder Raina, Shilpa Sood

**Affiliations:** 1 Division of Veterinary Pharmacology and Toxicology, Faculty of Veterinary Science and Animal Husbandry, R S Pura, Jammu, 181102 India; 2 Division of Veterinary Pharmacology and Toxicology, Faculty of Veterinary Science and Animal Husbandry, R S Pura, Jammu, 181102 India; 3 Division of Veterinary Pharmacology and Toxicology, Faculty of Veterinary Science and Animal Husbandry, R S Pura, Jammu, 181102 India; 4 Division of Veterinary Pathology, Faculty of Veterinary Science and Animal Husbandry, R S Pura, Jammu, 181102 India

**Keywords:** Imidacloprid, Arsenic, Testes, Antioxidant, Oxidative damage

## Abstract

**Background:**

It is an established fact that humans and animals are exposed to more than one chemical concurrently from various sources such as food, air and water. In the past, much emphasis was laid on evaluating the toxic effects of a single chemical. Nowadays an increased attention is being paid to the interaction of xenobiotics with one another. Therefore, a study was aimed to evaluate the potentiating effect of imidacloprid (IMI) on arsenic-induced testicular toxicity in rats.

**Methods:**

Adult male Wistar rats randomly divided into eight groups with six in each were subjected to daily oral administrations for 28 days. Group I served as control, group II received IMI at the dose rate of 16.9 mg/kg body weight, group III, IV and V received arsenic at the dose rate of 50, 100 and 150 ppb in drinking water whereas group VI, VII and VIII received both arsenic and IMI.

**Results:**

Repeated oral administrations of IMI or arsenic (150 ppb) alone resulted in a significant (*P* < 0.05) elevation in the levels of malondialdehyde (MDA) and advanced oxidation protein product (AOPP) along with significant (P < 0.05) decline in total thiols and antioxidant enzymatic activities indicating reduced antioxidant defense in testicular tissue of exposed rats. These findings were further corroborated with histological alterations in testes like fluid accumulation in interstitial spaces in IMI administered rats. Similarly, rats provided access exclusively to arsenic-containing drinking water induced degenerative changes in seminiferous tubules in a concentration-dependent manner. Concurrent administration of IMI and arsenic produced more severe antioxidant and histopathological alterations of testes as compared to exposure to either toxicant.

**Conclusions:**

Reduced antioxidant activities, increased MDA and AOPP levels with severe histopathological alterations in testes of rats on concurrent exposure indicated that IMI potentiated the arsenic-induced testicular toxicity in Wistar rats.

## Background

The ability of the chemical pollutants to affect reproductive health has garnered significant attention in recent decades and is further compounded by the endocrine disruptive chemicals in the environment [1–3]. Chemicals that disrupt normal endocrine functions may interfere with the hormonal pathways responsible for the control of reproduction [4] triggering morphological and functional abnormalities in reproductive organs. Testicular toxicity of insecticides and other toxicants result in male infertility in mammals [5].

Various experimental studies have shown cellular and molecular aspects of gonadal damage in animals during spermatogenesis following chemical exposure including insecticides [5, 6]. Imidacloprid (IMI), a newer neonicotinoid has gained wide acceptance as an insecticide due to its extremely high insecticidal potency, broad insecticidal spectra, excellent systemic properties in pests and a low mammalian toxicity [7]. Agrochemicals along with heavy metals from emissions and groundwater are the main sources responsible for the environmental contamination [8]. Among heavy metals, Arsenic (As) is categorized as a ubiquitous trace element and is the 52^nd^ most abundant element in the earth’s crust. Arsenic finds a place in the priority list of hazardous substances published by the Agency for Toxic Substances and Disease Registry [9]. Studies revealed that arsenic interacts with sperm DNA which may contribute to the mechanisms for a trans-generational reproductive effect [9, 10].

The concurrent exposure of more than one environmental toxicant in mammals induce depletion in antioxidant defence system as indicated with reduced levels of total thiols (TTH) including reduced glutathione (GSH). Antioxidant defence system scavenges reactive oxygen/nitrogen species (ROS/RNS) generated in tissues and sub-cellular compartments during intoxication [11]. It has been reported that excessive generation of these free radicals in testicular tissue reduces antioxidant defence resulting in the disruption of the functional integrity of membrane structures of mitochondria and other cytoplasmic organelles through peroxidation of phospholipids, proteins and nucleotides [12, 13]. Hence, ROS plays an important role in the pathogenesis of male reproductive system due to the existence of high polyunsaturated fatty acid (PUFA) in the testes which makes it more sensitive to oxidative insults [14, 15]. Therefore, the present study was aimed to evaluate the potentiating effect of imidacloprid (IMI) on arsenic-induced testicular toxicity in male Wistar rats.

## Methods

### Experimental animals and chemicals used

The study was conducted on adult male Wistar rats (180–200 g, 12-14 weeks of age) procured from Indian Institute of Integrative Medicine (IIIM), Jammu. The animals were provided standard pelleted ration and tap water for drinking ad-libitum. All animals were maintained under standard managemental conditions (22 ± 3 °C, 50–60% relative humidity and 12 h light-dark cycles). Prior to the start of the experiment, Wistar rats were acclimatized in the laboratory conditions for a period of 15 days. All the experimental animals were kept under constant observation during the entire period of study. The experimental protocol was dully approved by Institutional Animal Ethics Committee (IAEC) vide letter no AU/ANN/13–14/IAEC/143–54, dated 24/05/2013. Imidacloprid (17.8% SL) used in the present study was commercially obtained from Mahindra and Mahindra Ltd. Agribusiness, Mumbai, India. Analytical grade Sodium Arsenate (Product no. 20237) procured from S.D. Fine-Chem. Limited, Mumbai was used as a source of arsenic. All other chemicals used in the study were of analytical grade and purchased from different standard firms.

### Experimental protocol

The low observed adverse effect level (LOAEL) dose (16.9 mg/kg) of imidacloprid was used in the study to determine its effects alone and in conjunction with the different doses of arsenic in drinking water. As per the WHO guidelines, the maximum contaminant level (MCL) of arsenic in drinking water is 50 ppb [16] and in the present study, three dose levels viz. 50, 100 and 150 ppb in drinking water were used in the study [4.165 mg of Sodium arsenate used provided 1 mg arsenic].

Adult male Wistar rats were randomly allocated into eight groups with six rats each. Group I received distilled water (1 ml/day) and served as the control, whereas group II received IMI orally at the dose rate of 16.9 mg/kg body weight. Group III, IV and V were provided access exclusively to drinking water containing arsenic at the rate of 50, 100 and 150 ppb, respectively. The groups VI, VII and VIII received combined administration of IMI and arsenic at the dose rate of 16.9 mg/kg + 50 ppb, 16.9 mg/kg + 100 ppb and 16.9 mg/kg + 150 ppb, respectively. The animals received daily dosing of IMI between 9.00–10.00 AM daily for a period of 28 days. All animals were weighed weekly for modifying the total dose of IMI to be administered. The animals were observed for clinical signs if any, during the entire period of study.

### Collection and processing of samples

After the end of 28 days of daily administrations of toxicants animals were sacrificed by cervical dislocation and testes 1 g each was collected in 10 ml ice-cold 0.5 M Phosphate buffer (pH 7.4) and formal saline (10%) for antioxidant parameters and histopathological studies, respectively. Tissue homogenate (10%) was prepared by homogenizing the testicular tissue using Teflon coated homogenizer at 1000 rpm for 5–7 min at 4 °C.

### Determination of lipid peroxidation and protein oxidation product in testes

Levels of malondialdehyde (MDA) and advanced oxidation protein product (AOPP) were determined in the testicular tissue to determine the intensity of oxidation of membrane lipids and cellular proteins, respectively the following exposure to toxicant alone and in combination [17, 18].

### Determination of antioxidant parameters in testes

Total thiols (TTH) level was determined in testes as per the standard method using reduced glutathione and was expressed in mM [19]. Various antioxidant enzymes in testicular tissue viz., catalase (CAT), superoxide dismutase (SOD), glutathione peroxidase (GPx), glutathione reductase (GR) and glutathione-s-transferase (GST) were determined spectrophotometrically (UV-1601, Shimadzu) to assess the alterations in antioxidant system following exposure to toxicant alone or in combination [20–24].

### Histopathological studies

The histopathological studies were carried out according to standard methods. Formalin-fixed testes of the different group were embedded in paraffin, sectioned, stained with hematoxylin and eosin and examined under a light microscope for histopathological studies.

### Statistical analysis

The biochemical and oxidative stress parameters were analyzed for analysis of variance at 5% level of significance using the Duncan Multiple Range test (SPSS 16.0).

## Results

Antioxidant biomarkers in testicular tissue following repeated oral administration of IMI and arsenic alone and in combination in rats is presented in Table 1.

**Table 1:** Activities of various antioxidant enzymes and total thiols (TTH) level in testes of Wistar rats following repeated oral administrations of IMI and arsenic alone and in combination

Groups	TTH	GST	GR	GPx	SOD	CAT
I. Control	4.76^c^ ± 0.540	59.03^f^ ± 3.34	25.56^f^ ± 1.88	26.89^e^ ± 2.99	196.80^d^ ± 13.12	1694.41^e^ ± 62.69
II. Imidacloprid (IMI) (16.9 mg/kg)	3.26^ab^ ± 0.105	38.65^d^ ± 2.83	15.72^bcd^ ± 1.73	17.26^cd^ ± 1.30	136.44^bc^ ± 14.02	1152.61^d^ ± 42.63
III. Arsenic (50 ppb)	3.96^bc^ ± 0.151	50.65^e^ ± 3.64	21.58^ef^ ± 1.44	19.68^d^ ± 1.19	160.96^cd^ ± 15.02	1256.72^d^ ± 58.52
IV. Arsenic (100 ppb)	3.52^ab^ ± 0.302	41.48^d^ ± 1.90	19.30^de^ ± 1.50	16.24^cd^ ± 1.24	146.71^bc^ ± 13.59	1189.93^d^ ± 51.57
V. Arsenic (150 ppb)	3.23^ab^ ± 0.341	30.50^c^ ± 1.83	16.51^cd^ ± 1.33	13.65^bc^ ± 0.923	135.89^bc^ ± 14.95	1020.15^c^ ± 32.86
VI. IMI + As (50 ppb)	2.88^ab^ ± 0.229	24.28^bc^ ± 1.73	13.75^bc^ ± 1.42	10.02^ab^ ± 0.806	128.90^bc^ ± 12.97	744.78^b^ ± 36.45
VII. IMI + As (100 ppb)	2.75^a^ ± 0.425	20.65^ab^ ± 1.30	11.59^ab^ ± 1.09	8.75^a^ ± 0.899	117.26^ab^ ± 12.86	687.69^b^ ± 33.43
VIII. IMI + As (150 ppb)	2.40^a^ ± 0.457	14.60^a^ ± 0.350	8.50^a^ ± 0.967	6.01^a^ ± 0.753	84.25^a^ ± 9.90	544.78^a^ ± 34.73

Values are given as mean ± SE of 6 animals unless otherwise stated

Values having different superscript (a,b,c,d,e,f) in a column are statistically different from one another at 5% level of significance

Values of TTH (total thiols) are expressed in μM

Values of GST (glutathione S transferase) are expressed in μmol of CDNB conjugate formed/ min/ g of tissue

Values of GR (glutathione reductase) are expressed nmol of NADPH/min

Values of GPx (glutathione peroxidase) are expressed in Unit/ g of tissue

Values of SOD (Superoxide dismutase) are expressed in Unit/ g of tissue

Values of CAT (Catalase) are expressed in μmol H_2_O_2_ decomposed/ min/ g of tissue

### Glutathione-S-transferase (GST)

A significant (*P* < 0.05) decrease in GST activity was observed (34.5%) after repeated IMI administrations for 28 days considering the activity of the control group as 100%. Similarly, there was significant (*P* < 0.05) fall in GST activity within group III (14.2%), IV (29.7%) and V (48.3%) compared to the control. Similar significant (P < 0.05) decrease in the activities of GST were observed in group VI (58.9%), VII (65.0%) and VIII (75.3%).

### Glutathione reductase (GR)

Significant (P < 0.05) decrease in GR activity was observed in IMI exposed animals (38.5%), as well as in groups III (15.6%), IV (24.5%) and V (35.4%) compared to control group. Such a significant fall in GR activity is also seen in combined exposed groups VI, VII and VIII (46.2–65.6%) as compared to the control group.

### Glutathione peroxidase (GP_X_)

GPx activity of testicular tissue had significantly (*P* < 0.05) declined in group II (35.8%), III (26.8%), IV(39.6%) and V (49.2%) as compared to following repeated oral exposure of toxicants. Similarly, GPx activity was profoundly declined in concurrent exposed groups (62.7–77.6%) compared to the control group.

### Catalase (CAT)

CAT activity testes had significantly (*P* < 0.05) declined in all groups exposed to toxicants either alone (IMI or Arsenic) or in combination (IMI with arsenic) as to compared control group. The decline in CAT activity was observed to be concentration dependent with arsenic (25.8–39.8%). Such decline was more profound (56.0–67.8%) in combined exposed groups.

### Superoxide dismutase (SOD)

A significant (*P* < 0.05) fall in SOD activity was observed in group II (30.7%), III (18.2%), IV (25.5%) and V (30.9%) as compared to control group. A similar decline in the activity of SOD was observed in different combined administered groups (34.5–57.2%).

### Total thiols (TTH)

A significant (*P* < 0.05) decrease in mean TTH level was observed in IMI or arsenic alone administered groups - II (31.5%), III (16.8%), IV (26.1%) and V (32.1%) in comparison to control group. However decrease in TTH level a significant (*P* < 0.05) decrease in TTH level was observed in Group VI (39.5%), VII (42.2%) and VIII (49.6%) compared to control group.

#### Testicular damage indicators

**Fig. 1:**
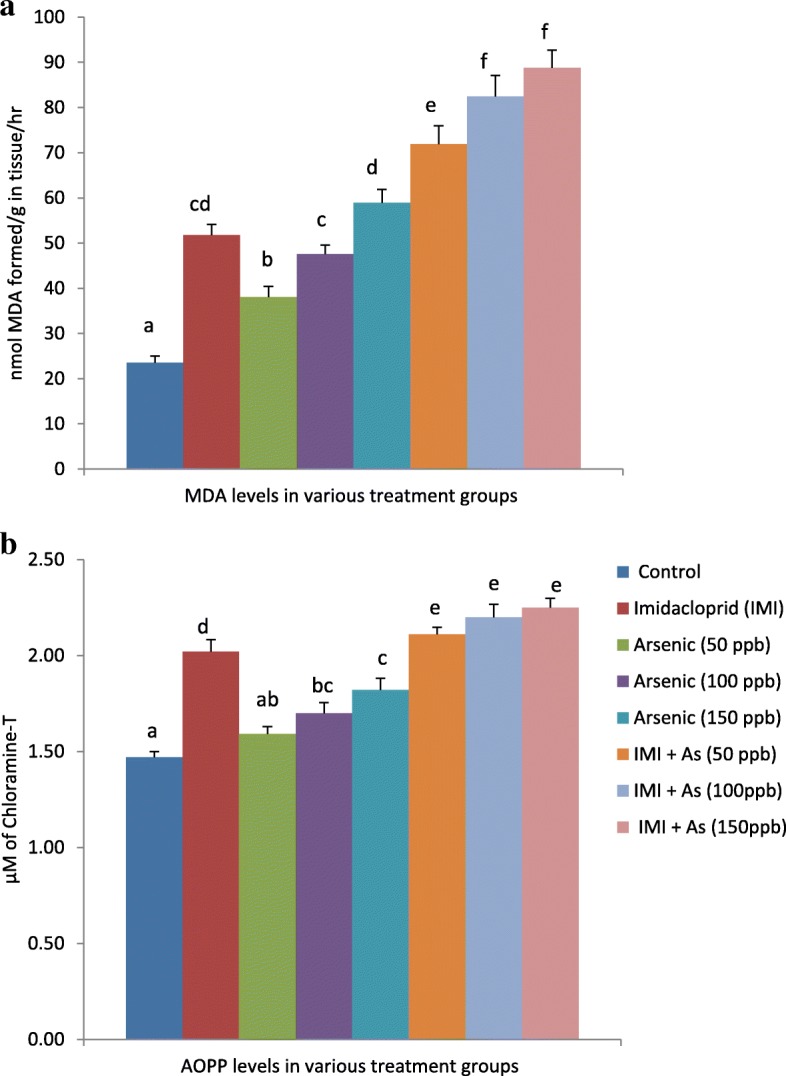
Effect of repeated oral administrations of IMI and arsenic alone and in combination on (**a**) MDA and (**b**) AOPP levels in testes of Wistar rats. (Mean values having different superscripts (**a**, **b**, **c**) are statistically different from one another at 5% level of significance)

MDA and AOPP levels in testes of different groups of animals exposed IMI or arsenic alone and in combination are shown in Fig. 1a and b respectively.

Significant (*P* < 0.05) increase in lipid peroxidation (MDA levels) were observed in group II (120.4%), III (62.0%), IV (102.5%) and V (151%) after repeated administration of IMI or arsenic for 28 days. However such rise was significantly higher (P < 0.05) in combined administered groups viz. VI (206.3%), VII (251.4%) and VIII (278.4%) compared to the control group.

A significant (P < 0.05) increase in protein oxidation (AOPP) levels was observed in IMI exposed group (37.4%) as compared to the control group. Arsenic in different used concentrations was less effective in increasing the AOPP levels (15.6–23.8%). However, increased AOPP levels were higher in co-exposed groups (43.5–53.1%).

### Histopathological alterations

**Fig. 2:**
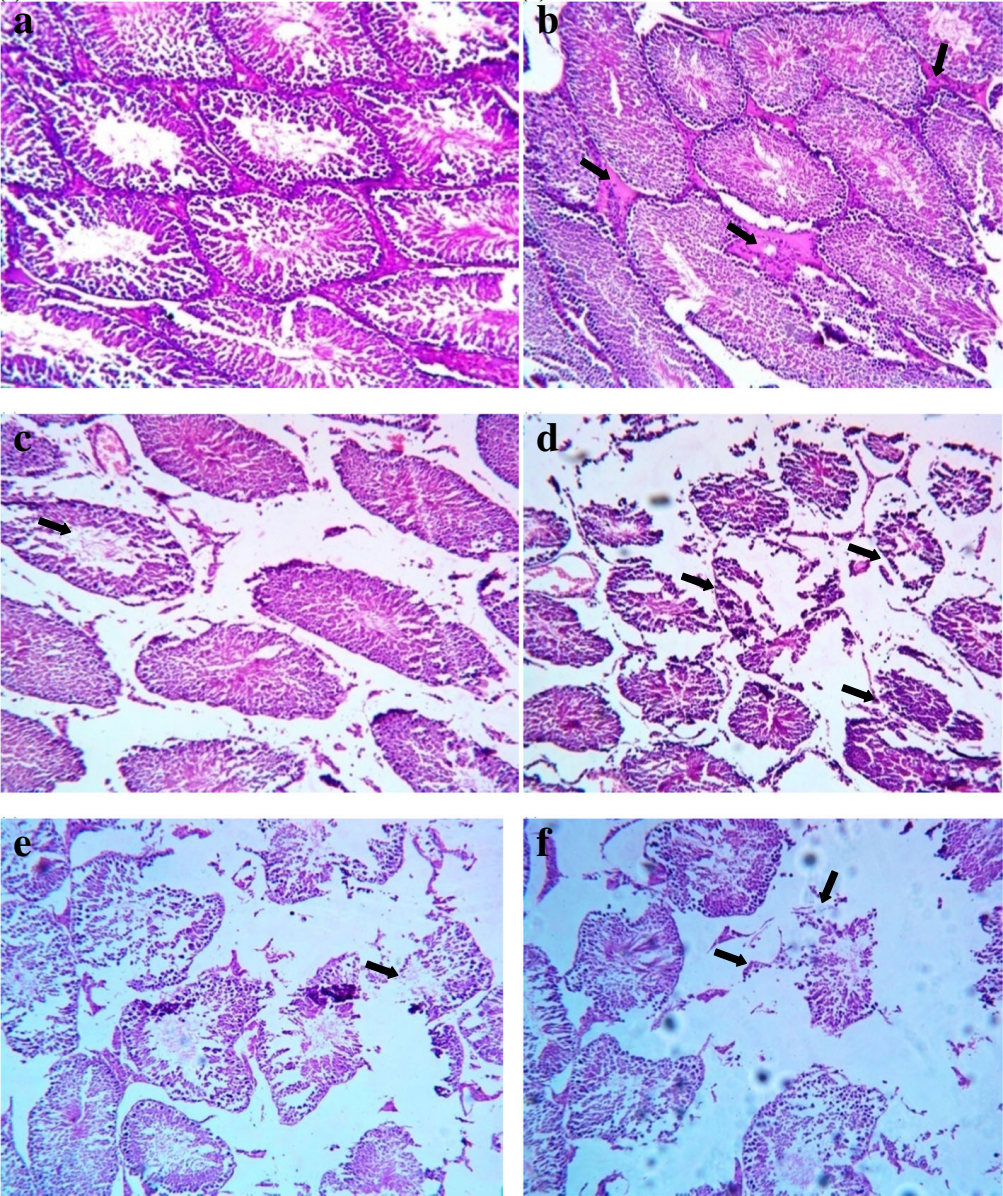
Photomicrograph of H & E (10X) stained sections of formalin fixed testes (**a**) Control: normal testicular parenchyma with seminiferous tubules and interstitial tissue (**b**) IMI administered group: mild edema in the interstitial spaces (**c**) Arsenic group: mild degeneration and necrosis in germ cells (**d**) group VI disruption of seminiferous tubules and necrosis of germ cells (**e**) Group VII: Severely necrotic seminiferous tubules and (**f**) Group VIII: fibrillar debris representing the remnants of seminiferous tubules of testes in Wistar rats

The histopathological alterations in the testes following repeated oral administrations of IMI and arsenic alone or in combination in Wistar rats are shown in Fig. 2. In normal rats, histologically the testes had normal morphological features comprising of seminiferous tubules with central tubular lumen surrounded by a basement membrane and no microscopic lesion of pathological significance was observed. All stages of spermatogenesis could be appreciably seen and mature spermatozoa, interstitial tissue contained Leydig cells, macrophages were present in the lumen of control animal testes. In IMI exposed group, the testicular parenchyma did not show any significant histological alterations when compared to control, other than mild edematous fluid accumulation in the interstitial spaces. Testes in rats belonging to group III had appreciable but only mild degenerative changes in few seminiferous tubules. In rats belonging to group V appreciable degenerative and necrotic changes of germ cells were observed and such changes were not seen in rats administered lower concentration of arsenic.

In co-exposed rats (group VI) severe degenerative changes in seminiferous tubules with their number reduced along with degeneration and depletion of germ cells of all the lineages was observed. Increased concentrations of arsenic in drinking water and IMI induced more severe histopathological changes in the testes. Severe testicular necrosis was seen in group VII characterized by tubular shrinkage, decreased lumen diameter along with generalized necrosis and depletion of germ cells. More severe changes were appreciable in group VIII where tubules became severely necrotic with only remnants of fibrillar necrotic debris seen in testicular sections.

## Discussion

Arsenic endemic areas groundwater is the major source of intoxication to mammals. Similarly, IMI is most commonly used neonicotinoid insecticide for pest and insects management in agricultural and animal practices. Therefore the coexistence of arsenic and IMI in the environment is reality and concurrent exposure to humans and animals could be potentially hazardous.

### Effect of repeated oral administrations of arsenic alone and in-conjunction with IMI on antioxidant parameters in testicular tissue

Free radicals like hydroxyl (OH^−^), superoxide (O_2_^·-^), nitric oxide (NO^·^), hydrogen peroxide (H_2_O_2_) are commonly generated by auto-oxidation processes and/or enzymatic metabolic reactions are scavenged by cellular antioxidant system [25]. Repeated oral administrations of arsenic alone or in-conjunction with IMI increased cellular metabolic reactions leading to increased generation of free radicals. The inability of enzymatic antioxidant components like CAT, SOD and GPx to scavenge these excessively produced free radicals during metabolism of toxicants may induce testicular damage as indicated by increased levels of lipid peroxidation (MDA) and protein oxidation (AOPP) product after exposure with IMI and arsenic in Wistar rats. The increasing concentration levels of arsenic in drinking water proportionately increase the AOPP and MDA levels in testes of rats [26–28].

Protein and non-protein thiols (-SH group) play a significant role in scavenging free radicals including ROS. Thiols protect cellular components from oxidative insults either by direct scavenging of the free radicals and/or acts as the substrate for various antioxidant enzymes like SOD, GPx and GST [29]. In the present study, a significant reduction in the level of TTH in testicular tissue of rats exposed either to arsenic alone at 150 ppb or in concurrently exposed groups may be primary contributor of oxidative insults in testicular tissue. The replenishment of GSH during oxidative stress can be achieved by either increasing its biosynthesis or recycling by the activity of GR [30]. In the present study, reduction in the activity of GR may be responsible for the failure in replenishment resulting in the reduced level of GSH which decreases the direct and indirect scavenging of free radicals during intoxication [30]. The reduction in the activity of enzymes utilizing glutathione as a substrate (SOD, GPx and GST) may be due to the reduced level of thiols. The decreased antioxidant enzymes have been reported to induce oxidative damage of cellular membranes of spermatozoa due to generated free radicals which inhibit their number and increase their abnormality rate [31, 32]. Arsenic exposure along with IMI administration to Wistar rats produced a more profound decrease in antioxidant enzymatic activities than the administrations of either IMI or arsenic which is in consonance with reported findings [26].

The increased free radicals interact with the cellular proteins and lipids leading to the peroxidation as indicated by increased levels of MDA and AOPP in rats administered with arsenic (150 ppb) alone or in the combination of Arsenic at various concentrations with IMI administered group. These results are in accordance with the reported findings on interaction studies of insecticides and metals [33–35]. Administration of both IMI and arsenic induce significant fall in tissue levels TTH as compared to the sole administration of toxicants. Co-exposure of rats to IMI and arsenic may have generated excessive free radicals in testicular tissue and its sub-cellular compartments which may have resulted in depletion of antioxidants, as indicated with decreased TTH level in testes [36].

The significant increase in the lipid peroxidation and protein oxidation product was observed testicular tissue of the IMI administered group. These observations have also been in agreement with the finding reported in the male reproductive system following administrations of insecticides [33, 37]. The exposure of arsenic through drinking water also induces a significant rise in MDA and AOPP levels in the testes of Wistar rats. Various studies have also reported similar results on chemical induced oxidative damage in testes [28, 38, 39]. Concurrent exposure of both the toxicants produced a more significant increase in lipid and protein oxidation levels as compared to individual administration of either toxicant. The testes are rich in PUFA and prone to lipid peroxidation [40]. Peroxidation of membrane lipids can result in disruption of cell structural integrity [41] leading to cellular damage. Hence, the increase in MDA and AOPP levels caused by both the toxicants in the rat testes suggests peroxidation of PUFA in testicular cells, which can cause impairment of normal testicular and sperm abnormalities [41].

### Histopathology alterations in testes

Microscopic examination of testes in rats of control group revealed normal mature seminiferous tubules with complete series of spermatogenesis and high spermatozoa concentration in the lumen. However, repeated oral administrations of IMI alone in rats showed progressive congestion in blood vessels and mild oedema in the interstitial space. IMI and arsenic in drinking water induced mild to severe degenerative changes of germ cells in testes of Wistar rats [34]. Increased concentration of arsenic along with IMI induced severe testicular necrosis characterized by tubular shrinkage, decreased lumen diameter along with generalized necrosis and depletion of germ cells. More severe changes were appreciable in animals co-exposed with arsenic at 150 ppb along with IMI made tubules severely necrotic with only remnants of fibrillar necrotic debris seen in testicular sections. The pronounced histopathological alterations are further corroborated by the increased levels of MDA, AOPP and reduced TTH and antioxidant defence increased the testicular tissue damage in Wistar rats. Similarly, another insecticide like acetamiprid administration produced vacuolization of the seminiferous tubules with a reduced number of spermaids and interstitial Leydig cells in the testes of Wistar rats [42]. These results were similar to the effects of β-cypermethrin in male mice [43]. However, α-cypermethrin has been reported to induce oedema between the seminiferous tubules and vacuolization of the tubules in male mice [44, 45].

Arsenic exposure has been reported to produce ruptured follicles and few follicles showed a reduction in the number of spermatozoa in the testes of male albino rats [46]. In present study arsenic along with IMI showed severe degenerative changes in seminiferous tubules, tubular shrinkage, decreased lumen diameter with generalized necrosis and depletion of germ cells which became more prominent with the increasing concentration of arsenic doses in the combination groups and such observed alterations are in agreement with reported findings [32, 33, 47].

## Conclusion

The finding of the present study suggests that repeated oral administrations of arsenic or IMI alone produce testicular damage in male Wistar rats. Reduced antioxidant enzymatic activities, increased MDA, AOPP levels and severe histopathological alterations in testes observed following concurrent repeated exposure indicated that IMI potentiated the arsenic-induced testicular toxicity in Wistar rats.

## Abbreviations

AOPP: Advanced oxidation protein product
CAT: Catalase
GPx: Glutathione peroxidase
GR: Glutathione reductase
GST: Glutathione-s-transferase
IMI: Imidacloprid
MDA: Malondialdehyde
SOD: Superoxide dismutase
TTH: Total thiols
